# Peripheral Eosinophil Count May Be the Prognostic Factor for Overall Survival in Patients with Pancreatic Ductal Adenocarcinoma Undergoing Surgical Treatment

**DOI:** 10.3390/biomedicines12112596

**Published:** 2024-11-13

**Authors:** Wojciech Ciesielski, Izabela Kupryś-Lipińska, Anna Kumor-Kisielewska, Oliwia Grząsiak, Julia Borodacz, Sebastian Niedźwiecki, Piotr Hogendorf, Adam Durczyński, Janusz Strzelczyk, Alicja Majos

**Affiliations:** 1 General and Transplant Surgery Department, Medical University of Łódź, 90-419 Łódź, Poland; wojciech.ciesielski@umed.lodz.pl (W.C.); oliwia.grzasiak@umed.lodz.pl (O.G.); piotr.hogendorf@umed.lodz.pl (P.H.); adam.durczynski@umed.lodz.pl (A.D.); janusz.strzelczyk@umed.lodz.pl (J.S.); 2 Internal Medicine, Asthma and Allergy Department, Medical University of Łódź, 90-419 Łódź, Poland; izabela.kuprys-lipinska@umed.lodz.pl; 3 Department of Pneumology and Allergy, Medical University of Łódź, 90-419 Łódź, Poland; anna.kumor@umed.lodz.pl; 4 Students’ Scientific Association in General and Transplant Surgery Department, Medical University of Łódź, 90-419 Łódź, Poland; julia.borodacz@student.umed.lodz.pl; 5 Department of Surgical Oncology, Medical University of Łódź, 90-419 Łódź, Poland; sebastian.niedzwiecki@umed.lodz.pl

**Keywords:** cancer biology, clinical observations, immunology, pancreatic ductal adenocarcinoma, pancreatic cancer

## Abstract

(1) Background: The importance of total eosinophil count in peripheral blood (EOS) as a type 2 inflammation marker is known to be fundamental in asthma, chronic sinusitis, and vasculitis. In cancer, despite their questionable antiproliferative effect, their role remains unclear. Our purpose was to describe the relationship between baseline blood EOS and overall survival (OS) in pancreatic ductal adenocarcinoma (PDAC) patients. (2) Methods: We retrospectively analyzed data from 137 adult patients who underwent surgical treatment for pancreatic ductal adenocarcinoma (PDAC) between the years 2012 and 2019. Patients with no recent history of systemic steroid use and without intraoperative metastases were included. Patients were categorized into two groups based on EOS (≥0.1 G/l and <0.1 G/l). Survival outcomes were analyzed using Cox proportional hazards regression models. (3) Results: According to EOS and PDAC stage, median OS values were as follows: in stage I–III, EOS ≥ 0.1 G/l group: 14.5 months, in stage I–III, EOS < 0.1 G/l group: 8.0 months, in stage IV, EOS ≥ 0.1 G/l group: 7.0 months, and in stage IV, EOS < 0.1 G/l group: 5.0 months. EOS < 0.1 G/l (vs. ≥0.1 G/l) was an independent prognostic factor for OS in both the uni- and multivariate Cox regression, respectively (HR = 1.48, *p* = 0.035 and HR = 1.57, *p* = 0.021). (4) Conclusions: Peripheral eosinophilia seems to be a potential independent prognostic factor. Further studies are necessary to confirm this hypothesis, since our findings suggest that type 2 inflammation may be the factor directly or indirectly lengthening the survival of patients with PDAC.

## 1. Introduction

The outcomes of pancreatic ductal adenocarcinoma cancer (PDAC) surgical resection have not increased substantially in the last decades. Five-year survival time median still does not exceed 20–30% with median overall survival (OS) up to 20 months [1]. The well-known predictive factors improving these outcomes are the administration of chemotherapy, pN0 nodal status, and R0 resection with a negative resection margin [2]. Despite this, researchers tried to find preoperative factors in peripheral blood, other than tumor markers (carbohydrate antigens), affecting the OS in patients with PDAC such as neutrophil-to-lymphocyte ratio (NLR), platelet-to-lymphocyte ratio (PLR), eosinophil–lymphocyte ratio (ELR), and FT3 to FT4 conversion ratio or gender-specific coagulation profile [3,4,5,6,7]. Current research commonly aims to find the mechanisms underlying the systemic inflammation caused by PDAC, which is probably related to the microenvironment of the tumor-causing immunologic disorder. This knowledge could lead to a better understanding of the pathogenesis of the disease and the development of patient-dedicated immunotherapies to improve the outcomes [8].

The human immune system is the guardian of our well-being—it recognizes pathogens and initiates a suitable response. Eosinophil is one of the elements of its arsenal. Eosinophils are leukocytes that play a role in the immunological responses of the organism by receptors for cytokines, chemokines, and adhesion molecules. It is involved in parasitic, bacterial, fungal, and viral infections as well as response to tissue damage and metabolic disorders. As their involvement is promoted by Th2 lymphocytes, eosinophilic inflammation is also named type 2 inflammation. In recent years, its role in the pathogenesis of various diseases has gained interest from researchers [9].

Eosinophils are regulated by adaptive immunity and treated as effector cells [9]. Under physiological conditions, there is high concentration of eosinophils in the gastrointestinal (GI) tract, where they regulate mucosal immunoglobulin A (IgA) secretion by producing interleukin 1 (IL-1), regulate the number of Th17 lymphocytes by expressing an antagonist for receptor of interleukin 1 (IL-1R), and regulate Treg differentiation through tumor growth factor β (TGF-β) and retinoic acid [10,11,12]. These processes take a role in maintaining the homeostasis between suppression and promotion of cell proliferation. IL-5 and eotaxin-1 (CCL11) are among the most important and effective eosinophil-specific chemokines targeting eosinophils, promoting its maturation in bone marrow, triggering the release of eosinophils into the blood and causing directed trafficking of eosinophils into tissues [13]. 

Following this biological pathway, Simson et al. in 2007 found out that eosinophils could have a potential antiproliferative effect in in vivo studies in mice as an effector cell in cancer immune surveillance—it suppressing in vivo and in vitro carcinogenesis and/or the development of chemically induced cancers [13]. A clear relationship between EOS and cancer development is not well known, but EOS level has been already described as a prognostic factor for OS or progression-free survival in some cases of solid cancer tumors such as melanoma, colon cancer, neuroendocrine tumors, urothelial carcinoma, renal cell carcinoma, glioblastoma, xanthoastrocytoma, ovarian cancer, prostate cancer, leiomyosarcoma, non-small-cell lung cancer, and breast cancer [14,15,16,17]. Hypereosinophilia has also been described to be an early sign of the disease in patients with hematological cancer [18].

While the “eosinophilia” term refers to any EOS level above the normal range (usually 0–0.5 G/l), the exact definitions of eosinopenia vary between the publications, usually being defined as EOS near to 0 G/l, e.g., 0–0.05 G/l or 0.01 G/l. Hypereosinophilia, a state of too-high blood eosinophilia, is described as mild (0.5–0.15 G/l), moderate (1.5–5.0 G/l), or severe (>5.0 G/l) [19,20]. Normal range should be located in between, but as the knowledge about eosinophilic diseases evolves and the criteria for anti-eosinophilic treatment follow, “the norm” conception becomes elusive and uncertain. The authors discussed whether the optimum criterium for anti-eosinophil treatment is EOS > 0.3 G/l or 0.15 G/l. Also, eosinopenia is usually considered within the normal EOS range. There are two anti-eosinophil monoclonal antibodies accepted by the FDA in common use: mepolizumab (directly binds to circulating IL-5 and reduces the number of eosinophils) since 2015 and benralizumab (against the interleukin 5 α receptor, causing eosinopenia) since 2018; both generally well-tolerated, with no apparent association between treatment and malignancies [20,21,22]. However, it is worth noting that due to the short observation time, it should not be taken as the final conclusion. What is also known from the anti-eosinophil treatment studies, EOS reflects the intensity of local T2 inflammation only partially; so eosinopenia can be considered a sign that T2 inflammation not being present in the body. Interestingly, when pancreatic resection takes place, the tumor is removed along with surrounding tissues; if the local T2 inflammation would play a role in the biological course of the disease, the difference in OS should not be observed in the eosinopenic group of resectable patients. An extreme situation, where local inflammatory conditions influence peripheral EOS, is paraneoplastic hypereosinophilia, described also in PDAC cases. It is known in its obvious form (EOS > 1.5 G/l) to be rare, but this phenomenon is not fully understood [23]. Therefore, our aim was to assess the prognostic value of EOS measured directly before surgical treatment in the group of patients with PDAC, with special attention paid to the disease stage.

## 2. Material and Methods

We have analyzed retrospectively the data from the electronic medical record system of 137 adult patients, who consecutively underwent surgical resection of PDAC in the General and Transplant Surgery Department (Medical University of Łódź, Łódź, Poland) between the years 2012 and 2019 and fulfilled inclusion (no history of systemic steroid use at least 1 month before surgical resection, available: age, sex, blood morphology baseline with white blood cell differential, tumor localization, grade, stage: localized or regional, PDAC confirmation in the post-operational histopathological examination, survival time) and exclusion criteria (distant metastases found intraoperatively; neoadjuvant chemotherapy; diagnosis or being under diagnostic process of fungal or parasitic infection, eosinophilic asthma, hypereosinophilic syndrome or EGPA, use of systemic steroids treatment at least 1 month before surgery). All the patients were qualified for surgical procedure treatment without preoperative diagnosis (due to the tumor size or diagnostic failure of preoperative percutaneous biopsies)—including locally advanced stages where the priority was to obtain a tissue sample.

Samples of peripheral blood for blood morphology tests were collected on admission to the hospital (up to 24 h before surgery). Eosinophil counts were obtained as part of routine preoperative blood tests performed up to 24 h before surgery, so the “EOS” term in this study always refers to baseline blood tests. Blood samples were processed using an automated hematology analyzer (Sysmex XN-2000, Warsaw, Poland), which provided a complete blood count (CBC) with differential, including the absolute eosinophil count. The results were reported as absolute eosinophils per liter (G/l), and patients were categorized based on eosinophil levels into two groups: EOS ≥ 0.1 G/l and EOS < 0.1 G/l, according to the EOS median in I–III stage group.

The patients were classified according to the American Joint Committee on Cancer (AJCC) 8th edition TNM staging system, which is commonly used to assess the extent of pancreatic cancer. This staging system is based on three key factors [19]:

- T (Tumor): The size and extent of the primary tumor.
- N (Nodes): The involvement of regional lymph nodes.
- M (Metastasis): The presence of distant metastasis.

We categorized patients into two major groups:

- Stage I–III: This group included patients with resectable or borderline resectable disease. These patients were eligible for curative surgical resection, either through pancreaticoduodenectomy (for tumors located in the head of the pancreas) or distal pancreatectomy with splenectomy (for tumors located in the body or tail of the pancreas).
- Stage IV: This group comprised patients with unresectable or locally advanced disease. These patients either underwent palliative surgery or biopsy depending on the extent of the disease at the time of surgery.

Extreme values of baseline peripheral eosinophils count >0.4 G/l constituted 3.6% of all observations in terms of tested clinical data, these patients were analyzed along with others in U tests and survival analysis. 

We have decided not to involve patients with PDAC undergoing surgical resection after 2019 to ensure a sufficiently long follow-up as well as in purpose to avoid the influence of possible COVID-19 on the calculations.

Statistical analyses were performed using Statistica 13.1 software. Nominal variables were compared using the chi-square test, while continuous variables were analyzed with the Mann–Whitney U test. Survival medians were estimated through the Kaplan–Meier method. In the analysis of subgroups, differences in survival curves were evaluated with the log-rank test or its equivalent for multiple-group comparisons. Cox proportional hazards regression was applied for survival analysis. All tested nominal parameters were included in uni- and multivariate analyses. Statistical significance was defined as *p* < 0.05 for all tests.

In our study, the variables selected for univariate and multivariate analyses were chosen based on their potential prognostic relevance in PDAC, as supported by prior research. Specifically, we included EOS, tumor stage, and other clinical parameters due to their established or hypothesized roles in cancer prognosis. For example, the role of eosinophils in inflammation and immune response has been suggested as significant in various malignancies, with low EOS linked to poorer outcomes in solid tumors. The tumor stage is also well-recognized as a critical determinant of patient survival in PDAC. By including these factors, our analysis aimed to explore how each independently and collectively impacts overall survival, providing a comprehensive assessment of their prognostic value in PDAC.

## 3. Results

### 3.1. General Characteristics of the Study Group

The study group consisted of n = 65 (47.4%) I–III stage and n = 72 IV(52.6%) stage IV subjects, with a total number of study participants n = 137 (general characteristics of the study group are presented in Table 1). PDAC’s grade, localization, and patients’ sex, age, EOS, and relative eosinophilia levels did not differ statistically significantly between the groups of stage I–III and IV, while 6-, 12-, and 24-month survival percentages were higher in stage I–III group, as expected. We have chosen the median EOS in the I–III group (0.1 G/l) as the cut-off point complementary to our research hypothesis; it is worth mentioning that it is almost the same as the EOS median in the IV group (0.11 G/l) and exactly the same after rounding to one decimal place.

### 3.2. EOS Levels and Clinical Parameters

**Figure 1:**
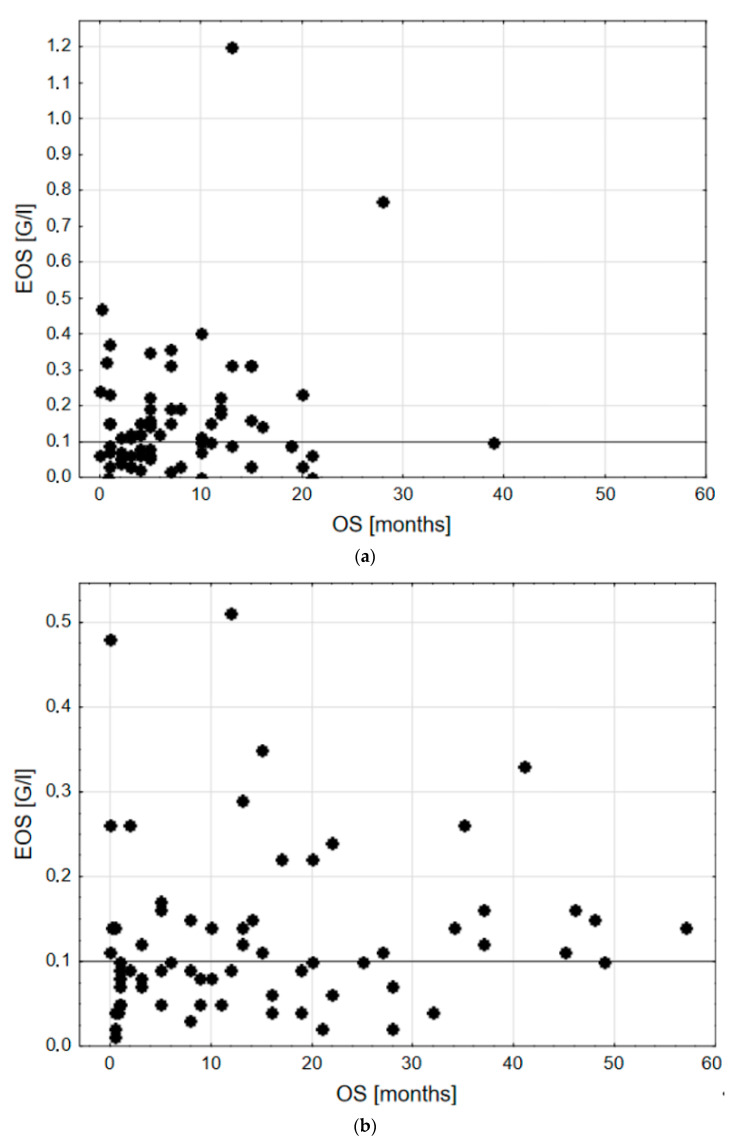
EOS vs. OS distribution in groups according to stage. (**a**) stage IV and (**b**) stages I–III. Cut-off point EOS 0.1 G/l was marked with a horizontal black line.

There were n = 58 subjects presenting baseline EOS < 0.1 G/l (42.3%) and n = 79 with EOS ≥ 0.1 G/l (57.7%). Subgroups according to EOS (<0.1 G/l vs. ≥0.1 G/l) did not differ statistically significantly in the main clinical features terms (Table 2). Relative eosinophilia seems to be a derivative of EOS in the group of patients with higher OS. Interestingly, 12- and 24-month survival was longer in EOS ≥ 0.1 G/l group. When comparing data distribution of EOS values vs. OS in different stages, in the IV stage group, no detectable relation between them can be found, while in the I–III group, there is a blank site visible on the chart; only one subject with OS > 30 months was noted in EOS < 0.1 G/l group (1.7%), whereas in EOS ≥ 0.1 G/l n = 10 such cases occurred (12.7%) (Figure 1). N = 2 cases of moderate hypereosinophilia in the stage IV group can be described as outliers, both in terms of EOS and OS values.

### 3.3. Analysis of Survival

EOS status was revealed to be a statistically significant OS predictor in Cox proportional hazard regression. Low EOS (<0.1 G/l) was a predictor of poor prognosis both in univariate (HR = 1.48; *p* = 0.035; Table 3) and multivariate (HR = 1.57; *p* = 0.021; Table 4) analyses. Additionally, stage IV was an independent poor OS predictor in both kinds of regression, respectively (HR = 1.88; *p* = 0.001 and HR = 1.73; *p* = 0.003). Other tested parameters did not reach statistical significance. 

**Figure 2:**
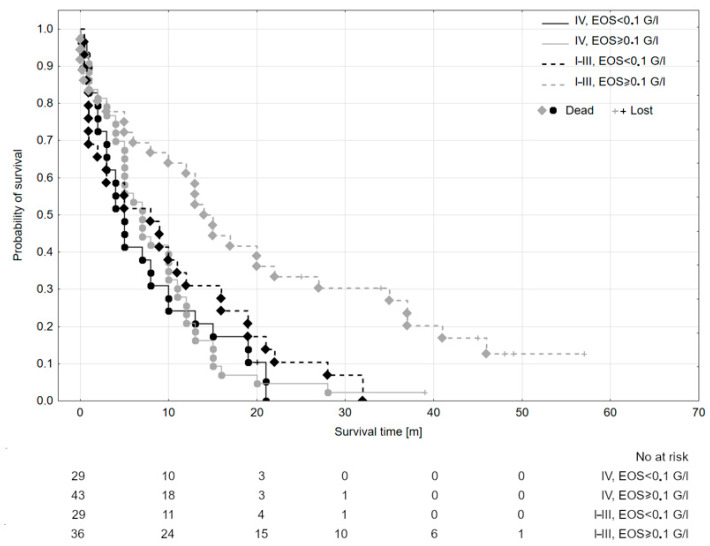
Kaplan–Meyer curves for subgroups according to the stage and baseline peripheral eosinophils count (G/l); *p* = 0,024. “IV” and “I–III” refer to the PDAC stage.

Dividing the whole study population according to two confirmed significant factors—stage and EOS status—led to distinguishing Kaplan–Meier curves for OS with *p* = 0.024 (Table 5 and Figure 2). Median survival times were decreasing with the increase in EOS and for disease advancement: for stages I–III and EOS ≥ 0.1 G/l: 14.5 months, for stages I–III and EOS < 0.1 G/l: 8.0 months; for stage IV and EOS ≥ 0.1 G/l: 7.0 months, for stage IV and EOS < 0.1 G/l: 5.0 months. The course of the curve for stages I–III and EOS < 0.1 G/ resembles the courses of curves for stage IV groups.

## 4. Discussion

### 4.1. EOS and the Risk of Malignancies or Death

**Figure 3:**
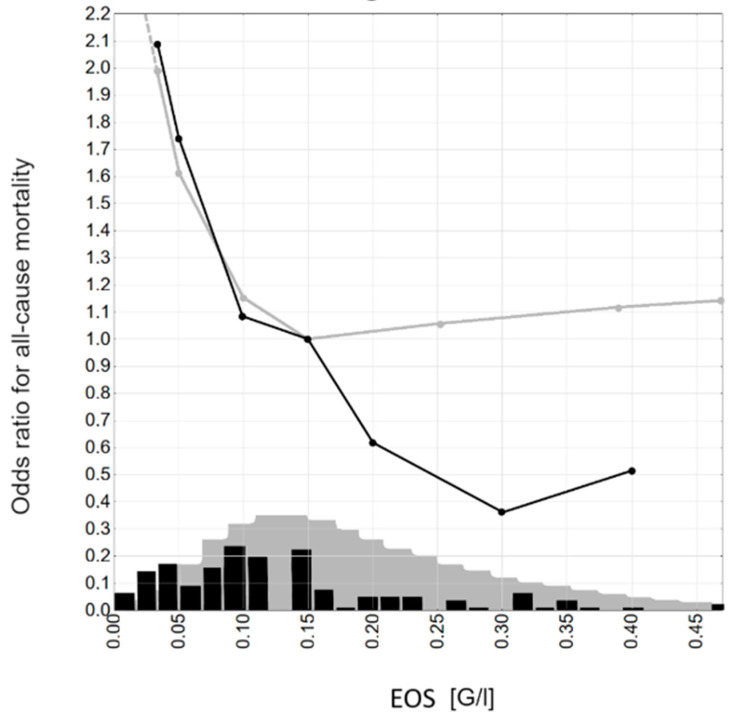
Odds ratios for all-cause mortality: black line—1-year all-cause mortality, current study; black histogram—blood eosinophilia values dispersion in the current study; grey lines—4-year all-cause mortality, Andersen et al.; grey histogram—blood eosinophilia values dispersion in Andersen et al. study; the histograms of blood eosinophilia are included for illustrative purposes—black for the current study and grey for Andersen et al. study [18].

Our results suggest a connection between low EOS and poor prognosis. Is it a specificity of PDAC or a more widely visible trend? Curran and Bertics in their publication regarding gliomas made some interesting observations: Firstly, immunity of atopic conditions, high total IgE, IL-4 activation, and, in consequence, eosinophil action may promote anticancer processes; thus, secondly, there were some data published that are in line with that hypothesis; allergy, asthma, and eczema diagnoses were associated with lower HR for glioma development (OR = 0.34–0.96, dependent on the study) [24]. Andersen et al. performed research on eosinophilia’s predictive value for solid cancer development in the Danish population. The researchers used The Copenhagen Primary Care Differential Count (CopDIFF) database containing morphology test results from 626,157 adults from 2000 to 2010 and randomly chose 356,196 for statistical analysis. They investigated the 3-year incidence of any solid tumor, which revealed that patients with eosinophils count between 0.5 and 1.0 G/l (mild eosinophilia) had 1.93 times higher risk of developing bladder cancer in comparison to no (<0.5 × 10^9^/l) or severe (≥1.0 × 10^9^/l) eosinophilia [18]. In another study, they assessed the risk of 4-year all-cause mortality based on blood eosinophilia, revealing that it is the highest for eosinopenia, the lowest for individuals with EOS of about 0.15 G/l, and for higher values, it increases proportionally to EOS level [24]. We have compared our results with the Andersen team’s findings (Figure 3). At the EOS level 0–0.1 G/l, the two OR trend lines seem identical, but with higher values, a reverse trend is visible for patients with PDAC.

### 4.2. TATE Phenomenon

As shown in Figure 2, in this research, the prognosis for patients with PDAC with lower stages but also low EOS is nearly as poor as in stage IV. The difference between stage groups in our study was resectability; this directs attention to potential local T2 inflammation. Few authors analyzed TATE (tumor-associated tissue eosinophilia) significance with inconsistent conclusions. Meta-analysis authored by Hu et al. [25] summarized them: TATE was described as protective in oral cancer [26,27], laryngeal cancer [28], nasopharyngeal cancer [29], colorectal cancer [30,31,32,33], esophageal cancer [34,35], Hodgkin’s lymphoma [36], gastric cancer [37], penile cancer [38], and cervical cancer [39]; without prognostic significance in oral cancer [40] and bladder cancer [41] and as a predictor of shorter OS in tongue cancer [42], head and neck cancer [43], laryngeal cancer [44,45], Hodgkin’s lymphoma [46,47], and cervical cancer [48]. Although the relationship between TATE and tumor-associated blood eosinophilia (TABE) is not yet determined in patients with solid malignancies, it seems reasonable to assume that as it is adopted in eosinophilic asthma, not necessarily TATE and TABE represent each other, but probably eosinopenia excludes TATE. There are no studies on TATE in patients with PDAC.

The observed differences in survival outcomes between patients in stages I–III and IV, as shown in Figure 2, may stem from differences in tumor resectability and immune environment across these stages. Patients in stages I–III generally undergo curative resection, allowing removal of both the primary tumor and, potentially, areas of type 2 inflammation that may exert local immunomodulatory effects. In contrast, stage IV patients typically present with unresectable or metastasized tumors, where systemic inflammation may not be as locally controlled, potentially leading to the more rapid disease progression and reduced survival observed. Additionally, the effects of tumor-associated eosinophilia may vary between localized and advanced disease, possibly due to differing levels of tumor immune interaction and the distinct biology of metastatic PDAC.

### 4.3. EOS in Malignancies During Treatment

The timing of baseline EOS measurement, as in our study, can be understood as a starting point or as one of many points in the continuum of PDAC illness. The second approach provides an interesting perspective; but as we lack data about further treatment of our cohort, we have to look into the published data regarding EOS during the treatment of malignancies. The association between EOS and OS was examined in several types of cancers.

Alves et al. described eosinophilia >0.5 G/l to be a positive predictive factor for better outcomes (non-progression) in patients with non-small-cell lung cancer undergoing immunotherapy. Hypereosinophilia was also associated with a higher risk of immune-related adverse effects such as asthenia, hypothyroidism, pneumonia, and pruritus [16]. 

In a group of patients with various cancers (including PDAC), it was shown that survival (OS) was higher in the group of patients who achieved EOS > 0.5 G/l on treatment. Each increase of 0.1 × 10^9^/l in the number of eosinophils was associated with a 28% increase in the chance of better disease control. Higher EOS was also associated with developing toxicity of any grade [49]. 

Ghebeh et al. investigated the association of eosinophil count with response to chemoimmunotherapy in advanced triple-negative breast cancer. Higher eosinophil count and increase in peripheral blood eosinophil count >0.3 G/l during the treatment were associated with better OS in this group of patients [17]. 

Simplifying these conclusions, as eosinophils are known for their destructive force, they may be involved in the cleaning process needed after effective systemic treatment (as in cited studies) or effective cytotoxic reactions (possible in our study cohort). Further research is needed to clarify this hypothesis.

### 4.4. EOS in Patients with PDAC

Despite the increasing number of reports on the role of eosinophilia in the development of cancer disease, PDAC is almost a blank page on this topic. The available literature is limited to some case reports [50,51,52,53,54] and retrospective studies by Holub [14], Abu-Shawer [54], and Ohkuma [19] (Table 6).

Holub et al. analyzed the records of patients who had undergone external beam radiotherapy; information about qualification for this regiment is lacking. The cut-off point adopted was EOS 0.5 G/l with <0.5 subgroup of n = 63 (95.5%) vs. ≥ 0.5 G/l n = 3 (4.5%) and applying this division led to no statistically important results in Cox proportional hazard regression for OS; while in progression-free survival, univariate Cox analysis EOS ≥ 0.5 corresponded to good prognosis, and the result was statistically significant (HR = 0.25, *p* = 0.024). Nevertheless, ELR (eosinophil to lymphocyte ratio), also analyzed by Holub et al., was revealed to be a predictor for OS: with shorter survival associated with ELR < 0.04 (univariate HR = 3.33, *p* = 0.001). This group constituted 13.6% of the whole study group and was characterized by EOS median = 0.0 G/l (range = 0.0–0.1 G/l), which de facto corresponds to eosinopenia, and its poor prognosis is consistent with our study findings [14]. The study of Abu-Shawer et al. concentrated on detecting metastases; the cut-off point was chosen to predict their presence; despite this fact, the results also seem to be in line with ours (worse prognosis for low EOS values) [54]. 

Ohkuma et al. reported that higher eosinophil counts and eosinophil-to-lymphocyte ratios were associated with improved survival in patients with stage II resectable PDAC. In contrast, our study examined the prognostic value of eosinophils across a broader range of stages (I–IV) and found that while eosinophilia (defined as EOS ≥ 0.1 G/l) was associated with better survival outcomes, the overall median survival times were shorter than those reported by Ohkuma et al. Several factors may account for this discrepancy:

- Patient Population: Our cohort included patients with both resectable (stage I–III) and unresectable (stage IV) PDAC, while Ohkuma et al. focused solely on resectable stage II patients. The inclusion of patients with advanced-stage disease in our study likely contributed to the overall shorter survival outcomes.
- Follow-up and treatment protocols: The duration of follow-up and the therapeutic approaches, such as adjuvant chemotherapy and radiation, varied between studies. In our cohort, patients with stage IV disease were more likely to have received palliative care, which could explain the shorter survival times observed. The specific treatment protocols followed by patients in the Ohkuma study were also not explicitly comparable to ours, potentially contributing to the differences in outcomes.
- Methodology: Differences in the statistical methodologies used may also explain the variation in survival data. While both studies used Cox proportional hazard models, the cutoff points for eosinophil levels and the variables included in the multivariate analyses differed. Ohkuma et al. used a cutoff of EOS < 0.126 G/l, while we used EOS < 0.1 G/l. Additionally, our study did not focus specifically on the eosinophil-to-lymphocyte ratio, which may further explain the differences in our results.

Despite these discrepancies, both studies reinforce the hypothesis that eosinophils may play a protective role in the immune response against pancreatic cancer. Our findings suggest that eosinophilia is associated with longer survival, particularly in earlier stages of PDAC, but further investigation is needed to fully understand the biological mechanisms underlying these observations [19]. Despite the shortcomings in methodology, all the papers report a similar distribution of EOS values as well as matching direction of low EOS prognosis; also, hypereosinophilic patients’ status seems to be unclear (Holub et al. and current study). None of the previous studies proposed a hypothesis explaining the observations. These results, together with Andersen et al. studies, strongly suggest that it is not blood eosinophilia per se, but the underlying mechanism may be responsible for the dismal prognosis associated with eosinopenia.

We believe that the difference in EOS significance between I–III and IV stages may result from EOS-related tumor suppression, which may be a phenomenon of significance restricted in time: initially, the influx of eosinophils to the tumor area stops vascularization, tumor spread, and immune recognition of the tumor cells, probably also fibrosis; but over time, this “dam” collapses, and the processes of destruction prevail. According to this hypothesis, early surgical intervention helps to remove the tumor not only *en bloc*, within the limits of macroscopically healthy tissue, but can also benefit from the “dam” of type 2 inflammation. This can also explain the finding from Figure 3; if we assume that in our study population, the reason for having higher EOS is just a proper reaction to the tumor cells, we do not expect that higher EOS will provide any better prospect; but it is not surprising that we see the damaging effect of not presenting this reaction.

In light of this hypothesis, it seems that even after resection, determining the status of preoperative eosinophilia may be potentially useful, especially if the underlying mechanisms are understandable. At this point, it has the potential to stratify the risk of short survival. Nevertheless, as the attempts to use the monoclonal antibodies—ipilimumab, pembrolizumab, durvulumab, and tremelimumab—have not yielded positive results [55,56,57,58,59,60], investigating T2 inflammation pathway will provide success in PDAC treatment, influencing different paths of immune reactions.

### 4.5. Study Limitations

The main disadvantage of this study is its retrospective character and following ambiguities, which surely influence the results; nevertheless, its results seem to correspond with available literature data.

Another limitation of our study is the lack of detailed data regarding important prognostic factors such as adjuvant therapy. Adjuvant chemotherapy is well-established as a critical component of post-operative management in PDAC, often contributing to improved survival outcomes. Unfortunately, due to the retrospective nature of the study and the lack of uniform documentation in patient records, we were unable to include information on adjuvant therapies in our analysis.

As such, we recommend that future studies integrate both surgical and adjuvant therapy data to provide a more comprehensive analysis of survival outcomes and prognostic factors in patients with PDAC. While retrospective analyses are often used as hypothesis-generating studies, we recognize that a prospective design would provide more reliable and controlled data. However, given the aggressive nature of PDAC and the relatively short survival time of many patients, conducting large-scale, long-term prospective studies remains a significant challenge.

## 5. Conclusions

Our study suggests that peripheral blood eosinophil count may serve as an independent prognostic factor for overall survival in patients with pancreatic ductal adenocarcinoma undergoing surgical resection. Higher baseline eosinophil levels seem to be protective, particularly in earlier stages of the disease. These findings highlight the potential role of type 2 inflammation in modulating the tumor microenvironment and influencing cancer progression. Further prospective studies are needed to confirm this relationship and explore the underlying mechanisms. Understanding these pathways may lead to the development of novel immunotherapeutic strategies that could improve survival outcomes in patients with pancreatic cancer.

## Figures and Tables

**Table 1:** Characteristics of the study group—general and according to the EOS level. *p* value refers to the stage I–III vs. stage IV comparison.

Parameter—Nominal	N, %	Stage I–III	Stage IV	*p*
		N, %	N, %	
Grade				0.743
1	26; 19.0%	11; 17.46%	15; 21.74%	
2	93; 67.9%	45; 71.43%	45; 65.22%	
3	18; 13.1%	7; 11.11%	9; 13.04%	
Tumor localization				0.537
Head	98; 71.5%	46; 73.02%	47; 68.12%	
Body/tail	39; 28.5%	17; 26.98%	22; 31.88%	
Stage		-	-	-
I–III	65; 47.4%			
IV	72; 52.6%			
Sex				0.644
Female	79; 57.7%	29; 46.03%	29; 42.03%	
Male	58; 42.3%	34; 53.97%	40; 57.97%	
Survival time				
6 months	58; 42.3%	40; 63,49%	34; 49,28%	0.100
12 months	74; 54.0%	31; 49.21%	17; 24.64%	0.003
24 months	5; 3.7%	14; 22.22%	1; 1.45%	0.000
Parameter—linear	Median; IQR			
Age, years	65.0; 59.0–70.0	66.0; 58.0–70.0	65.0; 61.0–71.0	0.149
EOS, G/l	0.11; 0.06–0.16	0.10; 0.06–0.15	0.11; 0.06–0.19	0.773
Relative eosinophilia, %	1.6; 0.8–2.5	1.57; 0.94–2.19	1.62; 0.72–2.53	0.773

**Table 2:** Characteristics of the subgroups according to baseline EOS level.

Parameter—Nominal	<0.1 G/l	≥0.1 G/l	*p*
	N, %	N, %	
Grade			0.207
1	9; 15.52%	17; 21.52%	
2	44; 75.86%	49; 62.03%	
3	5; 8.62%	13; 16.46%	
Tumor localization			
Head	40; 68.97%	58; 73.42%	0.568
Body/tail	18; 31.03%	21; 26.58%	
Stage			0.607
I–III	29; 50.00%	36; 45.57%	
IV	29; 50.00%	43; 54.45%	
Sex			0.227
Female	30; 51.72%	49; 62.03%	
Male	28; 48.28%	30; 37.97%	
Survival			
6 months	27; 46.6%	50; 63.29%	0.051
12 months	16; 27.6%	35; 44.30%	0.045
24 months	2; 3.45%	14; 17.72%	0.011
Parameter—linear	Median; IQR		*p*
Age, years	63.5; 58.0–70.0	66.0; 59.0–70.0	0.663
EOS, G/l	0.06 0.03–0.08	0.15; 0.12–0.26	0.000
Relative eosinophilia, %	0.76; 0.48–1.17;	2.35; 1.67–3.28	0.000

**Table 3:** Univariate Cox regression parameters.

Parameter	Effect Level–Reference Level	*p*	HR	HR 95%CI
Grade	1–3	0.177	0.53	0.28–1.00
	2–3	0.134	0.54	0.12–0.93
Localization	Head–body/tail	0.508	0.88	0.59–1.30
Stage	IV–I–III	0.001	1.88	1.29–2.75
Sex	Female–male	0.747	1.06	0.74–1.25
Age > 65 years	No–yes	0.414	0.86	0.60–1.23
EOS	<0.1 G/l–≥0.1 G/l	0.035	1.48	1.03–2.13

**Table 4:** Multivariate Cox regression parameters.

Parameter	Effect Level–Reference Level	*p*	HR	HR 95%CI
Grade	1–3	0.422	0.62	0.33–1.15
	2–3	0.126	0.57	0.32–0.99
Localization	Head–body/tail	0.427	1.18	0.79–1.76
Stage	IV–I–III	0.003	1.73	1.19–2.50
Sex	Female–male	0.933	1.01	0.71–1.45
Age > 65 years	No–yes	0.360	0.84	0.59–1.21
EOS	<0.1 G/l–≥0.1 G/l	0.021	1.57	1.07–2.31

**Table 5:** Survival time parameters according to stage and EOS.

Subgroup (Stage, EOS)	Median OS (m)	95% CI for Median OS (m)	No. of Complete Observations	No. of Lost Observations	n Total
I–III, EOS ≥ 0.1 G/l	14.50	8.0–22.0	30	6	36
I–III, EOS < 0.1 G/l	8.00	1.0–12.0	28	1	29
IV, EOS ≥ 0.1 G/l	7.00	5.0–10.0	42	1	43
IV, EOS < 0.1 G/l	5.00	3.0–8.0	28	1	29

**Table 6:** Characteristics of studies regarding EOS predictive value for OS in patients with PDAC.

Parameter	Holub	Abu-Shawer	Ohkuma	Current Study
Year	2020	2020	2021	2023
Protocol	Retrospective	Retrospective	Retrospective	Retrospective
n	66	355	67	137
Subjects with distant metastasis included?	No	Yes	No	No
Resection status known	Yes	No	Yes	Yes
Chemotherapy	Yes	N/A	N/A	N/A
Radiotherapy	Yes	N/A	N/A	N/A
EOS: median; mean (G/l)	0.1; 0.189	0.140; 0.190	N/A	0.11; 0.15
Cut-off point	0.5	0.143	0.126	0.1
Result from univariate Cox regression	≥0.5; HR 1.9 *p* = 0.300	≥0.143 HR = 0.9 *p* = 0.54	≥0.126 HR = 0.51 *p* = 0.042	<0.1: HR 1.48 *p* = 0.035

N/A—not available.

## Data Availability

Data will be shared after a reasonable request.
